# Effects of sepsis on venous microcirculation: non-invasive evaluation by quantitative near-infrared spectroscopy

**DOI:** 10.1186/cc9869

**Published:** 2011-03-11

**Authors:** V Papi, V Defraia, M Sandri, R Romano, R De Blasi

**Affiliations:** 1 Sapienza University of Rome, Italy; 2 Università Politecnica delle Marche, Ancona, Italy

## Introduction

Sepsis has several effects on microcirculation, including microthrombosis, interstitial edema and reduced reactivity of arteriolar tone leading to shunt areas [1]. Little is known about the effects of sepsis on the venous component of microcirculation. Changes of venular compliance and volume of the venular bed may affect cardiac preload, which has a key role in occurrence of cardiac failure. Near-infrared spectroscopy (NIRS) is a widely used, non-invasive technique that enables one to quantify the tissue oxyhemoglobin and deoxyhemoglobin (Hb) concentration, through which microvascular blood flow, compliance and oxygen consumption can be extrapolated [2]. The aim of our study was to evaluate the effects of sepsis on venous compliance and volume of the venular bed.

## Methods

Seven ICU patients with sepsis (according to ACCP/SCCM criteria [3]) and seven healthy subjects were studied. NIRS data were collected during several venous compressions at 20 to 30 to 40 mmHg. The venular bed volume increase at 20 mmHg was obtained from the total Hb concentration increase. Venular compliance was calculated as the volume increase and pressure inflated ratio. Results expressed as mean values ± SD for compliance and volume. The Mann-Whiney U test was performed to compare values in patients and controls.

## Results

The mean venular bed volume increase in the sepsis group was 3.32 ± 0.90 ml while in controls it was 7.80 ± 4.24 ml (*P *< 0.05). Venous compliance was significantly lower in the sepsis group compared with the control group (0.17 ± 0.06 ml/mmHg*l vs. 0.44 ± 0.10 ml/mmHg*l; *P *< 0.05).

## Conclusions

Sepsis affects the venous component of microcirculation by decreasing venular compliance and volume of the venular bed. This might be caused by a real decrease of venular bed volume, due to microthrombosis, or by an increase of venular tone. However, the clinical relevance of our findings is not known, and further studies are needed.
